# Navigating the future of diabetes: innovative nomogram models for predicting all-cause mortality risk in diabetic nephropathy

**DOI:** 10.1186/s12882-024-03563-5

**Published:** 2024-04-10

**Authors:** Sensen Wu, Hui Wang, Dikang Pan, Julong Guo, Fan Zhang, Yachan Ning, Yongquan Gu, Lianrui Guo

**Affiliations:** 1 Department of Vascular Surgery, Xuanwu Hospital, Capital Medical University, 45 Changchun Street, Xicheng District, Beijing, China; 2 Department of Intensive Care Medicine, Xuanwu Hospital, Capital Medical University, Beijing, China

**Keywords:** Diabetes, Diabetic nephropathy, Nomogram, All-cause mortality

## Abstract

**Objective:**

This study aims to establish and validate a nomogram model for the all-cause mortality rate in patients with diabetic nephropathy (DN).

**Methods:**

We analyzed data from the National Health and Nutrition Examination Survey (NHANES) spanning from 2007 to 2016. A random split of 7:3 was performed between the training and validation sets. Utilizing follow-up data until December 31, 2019, we examined the all-cause mortality rate. Cox regression models and Least Absolute Shrinkage and Selection Operator (LASSO) regression models were employed in the training cohort to develop a nomogram for predicting all-cause mortality in the studied population. Finally, various validation methods were employed to assess the predictive performance of the nomogram, and Decision Curve Analysis (DCA) was conducted to evaluate the clinical utility of the nomogram.

**Results:**

After the results of LASSO regression models and Cox multivariate analyses, a total of 8 variables were selected, gender, age, poverty income ratio, heart failure, body mass index, albumin, blood urea nitrogen and serum uric acid. A nomogram model was built based on these predictors. The C-index values in training cohort of 3-year, 5-year, 10-year mortality rates were 0.820, 0.807, and 0.798. In the validation cohort, the C-index values of 3-year, 5-year, 10-year mortality rates were 0.773, 0.788, and 0.817, respectively. The calibration curve demonstrates satisfactory consistency between the two cohorts.

**Conclusion:**

The newly developed nomogram proves to be effective in predicting the all-cause mortality risk in patients with diabetic nephropathy, and it has undergone robust internal validation.

## Introduction

Diabetes is a chronic, non-communicable, multisystemic disease characterized by elevated blood glucose levels due to the body’s inability to effectively produce or utilize insulin [1, 2]. According to statistics, in 2021, approximately 529 million people worldwide were affected by diabetes. It is estimated that by the year 2050, the number is expected to exceed 1.31 billion. While there are certain variations in the distribution of diabetes patients across different countries, it remains one of the primary causes of death and disability globally. This holds true regardless of national boundaries, age groups, or gender influences [3]. Prolonged elevated blood glucose levels can also lead to numerous secondary complications, affecting various systems or organs in the body. These complications include diabetic retinopathy, diabetic peripheral neuropathy, and diabetic nephropathy, among others [4].

Diabetic nephropathy (DN) is the most common complication of diabetes mellitus (DM), with estimates suggesting that as much as 40%-50% of diabetic patients may progress to diabetic nephropathy. Its primary characteristics include pathological albumin excretion in urine, glomerular lesions, and a decrease in glomerular filtration rate (GFR). Ultimately, it can lead to end-stage renal failure, associated with a higher mortality rate on a global scale [5, 6]. Furthermore, patients with DN often have concurrent cardiovascular diseases, which worsen the overall prognosis and further contribute to an increased mortality rate [7]. Epidemiological evidence indicates that as subsequent complications arise in individuals with diabetes, significant demands are placed on family, society, hospitals, and financial resources, and diabetes has emerged as a significant global public health concern [8].

Previously, several studies [9–11] have explored factors associated with the mortality rate of DN patients. However, effective tools for further predicting patient mortality have been lacking. Nomograms are a visual statistical prognostic tool that, by calculating scores based on potential predictive factors, can offer a rapid assessment of clinical risk stratification and prognosis. They are now widely used in the clinical assessment of disease prognosis [12, 13]. The aim of this study is to establish and validate an appropriate predictive model for the all-cause mortality rate in DN patients. This model is intended to rapidly identify high-risk patients and provide individualized interventions according to the patient’s situation timely, thereby helping to reduce the risk of premature death in individuals with diabetic nephropathy. The ultimate goal is to alleviate the burden on both families and the healthcare system. The study focuses on creating and validating a nomogram for predicting the all-cause mortality rate based on a United States population with diabetic nephropathy.

## Methods

### Database and study subjects

The National Health and Nutrition Examination Survey (NHANES) (https://wwwn.cdc.gov/nchs/) is a cross-sectional survey conducted by the Centers for Disease Control and Prevention (CDC) in the United States. It aims to assess the health and nutritional status of the non-institutionalized U.S. population. The survey employs a stratified, multistage probability design to recruit a representative sample of the U.S. population. Data is collected through structured interviews conducted at home, health screenings at mobile examination centers, and laboratory sample analysis [14]. NHANES is designed and managed by the National Center for Health Statistics, and its research ethics review is approved by its Institutional Review Board.

**Fig. 1:**
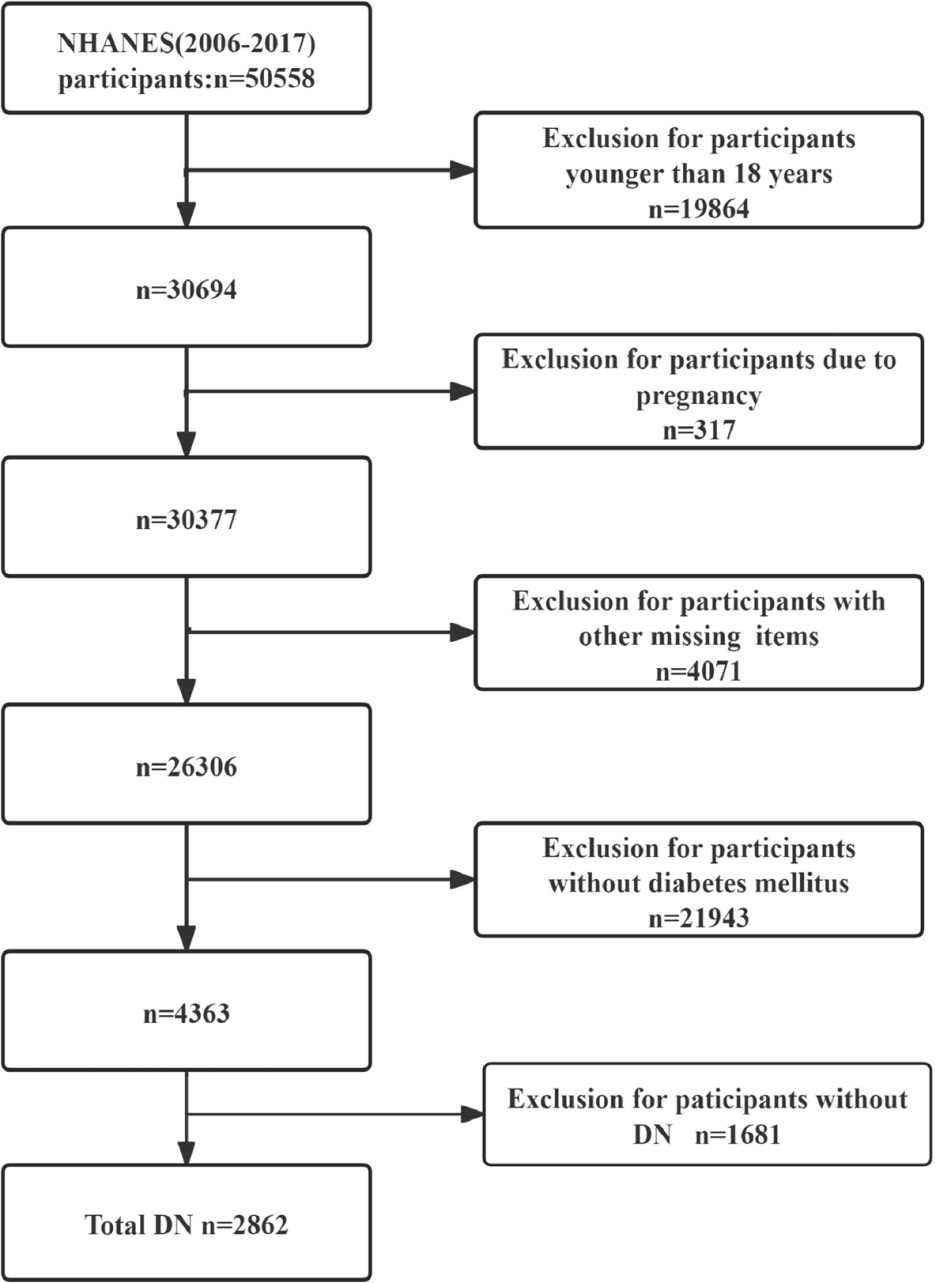
Flow chart of the cohorts

Participants included adult patients with diabetes, excluding those who were pregnant. Diabetes was defined as a diagnosis of diabetes and the use of insulin or oral hypoglycemic agents, with fasting blood glucose levels greater than 7.0 mmol/L (126 mg/dL) or glycated hemoglobin A1c (HbA1c) levels exceeding 6.5% [15]. The diagnosis of diabetic nephropathy was established in diabetic patients with an albumin-to-creatinine ratio (ACR) ≥ 300 mg/g or an estimated glomerular filtration rate (eGFR) < 60 ml/min/1.73m^2^ [16]. Participants lacking essential variable information were excluded. The detailed selection process is illustrated in Fig. 1. Follow-up all-cause mortality rates were determined using the national death index up to December 31, 2019.

### Potential predictors

When selecting variables in a prediction model, multiple factors should be considered comprehensively, including the correlation of variables, predictive ability, data availability, and model complexity. Standardized questionnaires were utilized to collect participants’ socio-demographic characteristics, smoking status, alcohol consumption, diabetes medication use, and hypertension information. Participants who smoked fewer than 100 cigarettes in their lifetime were classified as non-smokers, while those who had smoked over 100 cigarettes in the past but did not quit were defined as current smokers. Former smokers were individuals who had smoked over 100 cigarettes in the past but had already quit. Race/ethnicity was categorized as Mexican–American, other Hispanic, non-Hispanic white, non-Hispanic black, and other races. Educational attainment was classified as less than high school, high school or equivalent, and college or higher. Poverty Income Ratio (PIR) is calculated by dividing family income by poverty guidelines (specific to family size) and the appropriate year and state. This variable is an indicator of the ratio of household income to poverty scores. And the PIR were defined as < 1, 1–3, > 3. Body Mass Index (BMI) was calculated as weight divided by height squared (kg/m^2^) and categorized as < 25.0, 25.0–30.0, and > 30.0. We also included various laboratory markers, including hemoglobin, platelet count, urine creatinine, urine protein, total cholesterol, triglycerides, uric acid, creatinine, albumin, alanine aminotransferase, and aspartate aminotransferase, all of which were obtained from the NHANES database and measured as previously described in the literature [17, 18].

### Statistical analyses

Statistical analyses were conducted using SPSS (version 26) and R Studio (version R 4.2.2) software. Data collected from the NHANES database were randomly divided into training and validation sets in a 7:3 ratio, with subsequent comparison of data between the two groups. Categorical variables were presented as numbers and percentages, while continuous variables were expressed as mean ± standard deviation (SD). Chi-square test or Fisher’s exact test was employed for categorical variables, and t-test or Mann–Whitney U test for continuous variables. Initially, Cox univariate analysis was utilized to examine the impact of various factors on the survival time of patients in the training group. Concurrently, in the training cohort, LASSO logistic regression analysis was employed for multivariate analysis to identify independent risk factors. The factors selected by LASSO were further confirmed using COX multivariate regression model to identify meaningful predictors and construct the nomogram.

Once the nomogram was constructed, its performance was evaluated in both the training and validation groups. Discriminative ability of the nomogram was assessed using the area under the receiver operating characteristic (ROC) curve (AUC). Calibration curves were plotted to analyze the relationship between observed and predicted probabilities in both the training and validation cohorts. Additionally, a Decision Curve Analysis (DCA) method was employed to develop a model predicting the maximum net benefit [19]. Results with a *p*-value < 0.05 were considered statistically significant.

## Results

### Study population

After the final selection process, the study included a total of 2682 patients with diabetic nephropathy, with 1877 individuals in the training cohort and 805 individuals in the internal validation cohort. Apart from a difference in the incidence of stroke (*P* < 0.05), there were no statistically significant differences in other variables between the two groups. Median follow-up was 79 months, during the follow-up period, 532 (27.28%) individuals in the training cohort and 240 (29.81%) individuals in the validation cohort experienced all-cause mortality. Detailed characteristics data for both groups are presented in Table 1.

**Table 1:** Baseline characteristics in training and validation cohorts

Characteristic	Total *N* = 2682	Training Cohort *N* = 1,877	Validation Cohort *N* = 805	*P*
Gender				0.5
Male	1386(51.7%)	978 (52.1%)	408 (50.7%)	
Female	1296 (48.3%)	899 (47.9%)	397 (49.3%)	
Age	64.7 ± 12.1	65 ± 12	65 ± 12	0.729
Race				0.612
Mexican American	415 (15.5%)	290 (15.5%)	125 (15.5%)	
Other Hispanic	280 (10.4%)	195 (10.4%)	85 (10.6%)	
Non-Hispanic White	989 (36.9%)	677 (36.1%)	312 (38.8%)	
Non-Hispanic Black	776 (28.9%)	559 (29.8%)	217 (27.0%)	
Others	222 (8.3%)	156 (8.3%)	66 (8.2%)	
Education				0.834
Less than high school	959 (35.8%)	665 (35.4%)	294 (36.5%)	
High school diploma or GED	615 (22.9%)	435 (23.2%)	180 (22.4%)	
More than high school	1108 (41.3%)	777 (41.4%)	331 (41.1%)	
PIR				0.647
< 1	658 (24.5%)	461 (24.6%)	197 (24.5%)	
1–3	1311 (48.9%)	908 (48.4%)	403 (50.1%)	
> 3	713 (26.7%)	508 (27.1%)	205 (25.5%)	
BMI				0.513
< 25	361(13.5%)	247 (13.2%)	114 (14.2%)	
25–30	744(27.7%)	513 (27.3%)	231 (28.7%)	
> 30	1577(58.8)	1,117 (59.5%)	460 (57.1%)	
Smoke status				0.932
Never	869 (32.4%)	609 (32.4%)	260 (32.3%)	
Former	1473 (54.9%)	1,033 (55.0%)	440 (54.7%)	
Current	340 (12.7%)	235 (12.5%)	105 (13.0%)	
Drink	1184 (44.1%)	829 (44.2%)	355 (44.1%)	0.975
Hypertension	1960 (73.1%)	1,376 (73.3%)	584 (72.5%)	0.683
Heart failure	323 (12.0%)	229 (12.2%)	94 (11.7%)	0.703
CAD	575 (21.4%)	409 (21.8%)	166 (20.6%)	0.499
Stroke	292 (10.9%)	188 (10.0%)	104 (12.9%)	0.027
HbA1c %	7.40 ± 1.77	7.39 ± 1.78	7.41 ± 1.74	0.763
TC mmol/L	4.75 ± 1.21	4.72 ± 1.22	4.81 ± 1.19	0.094
TG mmol/L	2.18 ± 1.73	2.17 ± 1.78	2.22 ± 1.61	0.435
HDL mmol/L	1.24 ± 0.38	1.25 ± 0.39	1.22 ± 0.37	0.127
ALT U/L	26 ± 35	27 ± 40	25 ± 18	0.228
AST U/L	27 ± 25	27 ± 21	27 ± 32	0.884
Albumin g/L	41.2 ± 3.5	41.2 ± 3.6	41.0 ± 3.3	0.209
BUN mmol/L	6.54 ± 3.35	6.55 ± 3.42	6.50 ± 3.19	0.670
UA mg/dL	6.12 ± 1.65	6.11 ± 1.65	6.16 ± 1.64	0.452
ACR	241 ± 874	246 ± 914	227 ± 774	0.577
eGFR	52 ± 19	52 ± 19	52 ± 19	0.763
Follow-up, month	82.15 ± 37.73	82.07 ± 37.43	82.61 ± 36.31	0.836
Death	772 (28.8%)	532 (27.28%)	240 (29.81%)	0.594

Number and proportion were presented for categorical variables, mean and standard deviation were presented for continuous variables

*PIR* Poverty Income Ratio, *BMI* Body Mass Index, *CAD* Coronary Artery Disease, *HbA1c* Glycated Hemoglobin, *TC* Total Cholesterol, *TG* Triglyceride, *HDL* High-density lipoprotein, *ALT* Alanine aminotransferase, *AST* Aspartate aminotransferase, *BUN* Blood Urea Nitrogen, *UA* Uric Acid, *ACR* albumin-to-creatinine ratio, *eGFR* estimated glomerular filtration rate

### Development of nomogram

**Fig. 2:**
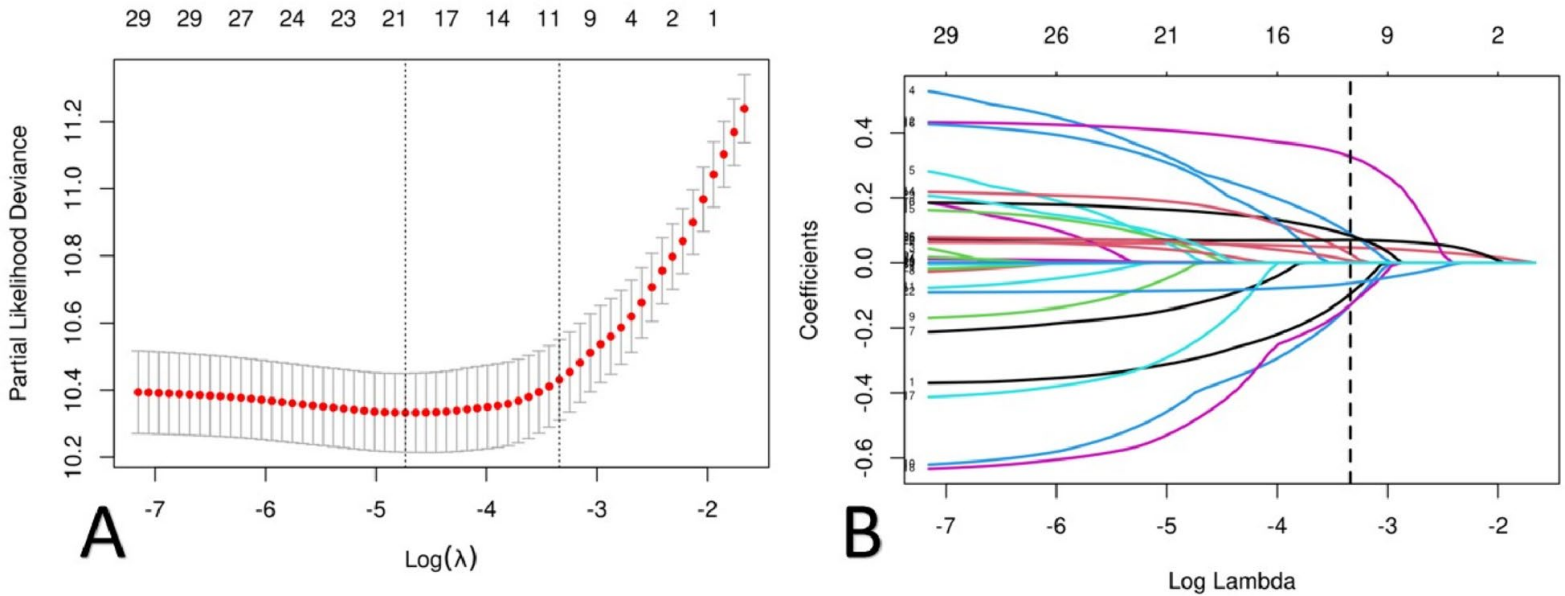
**A** Plot for LASSO regression coefficients. **B** Cross validation plot for the penalty term

We conducted a Cox univariate analysis on the variables in the training set of Table 1 to identify factors influencing survival. Simultaneously, we included the variables from the training set of Table 1 in a LASSO regression and, in combination with a multivariate Cox regression model (Table 2), ultimately determined 8 variables for the construction of a nomogram. The coefficients profile is plotted in the Fig. 2A and cross-validated error plot of the LASSO regression model is also shown in the Fig. 2B.

**Table 2:** Cox multivariate regression models in the training cohort

Characteristic	Live	Death	HR	95%CI	*p*
Gender					
Male	978	308	Ref	-	-
Female	899	224	0.65	0.55, 0.78	< 0.001
Age	1,877	532	1.07	1.06, 1.08	< 0.001
PIR					
< 1	461	135	Ref	-	-
1–3	908	289	0.83	0.67, 1.02	0.072
> 3	508	108	0.55	0.42, 0.71	< 0.001
Heart failure					
No	1,648	419	—	—	
Yes	229	113	1.74	1.41, 2.16	< 0.001
BMI					
< 25	247	105	—	—	
25–30	513	154	0.64	0.50, 0.82	< 0.001
> 30	1,117	273	0.51	0.41, 0.65	< 0.001
Albumin g/L	1,877	532	0.90	0.88, 0.92	< 0.001
BUN mmol/L	1,877	532	1.07	1.05, 1.09	< 0.001
UA mg/dL	1,877	532	1.07	1.02, 1.13	0.011

*HR* Hazard Ratio, *CI* Confidence Interval, *PIR* Poverty Income Ratio, *BMI* Body Mass Index, *BUN* Blood Urea Nitrogen, *UA* Uric Acid

**Fig. 3:**
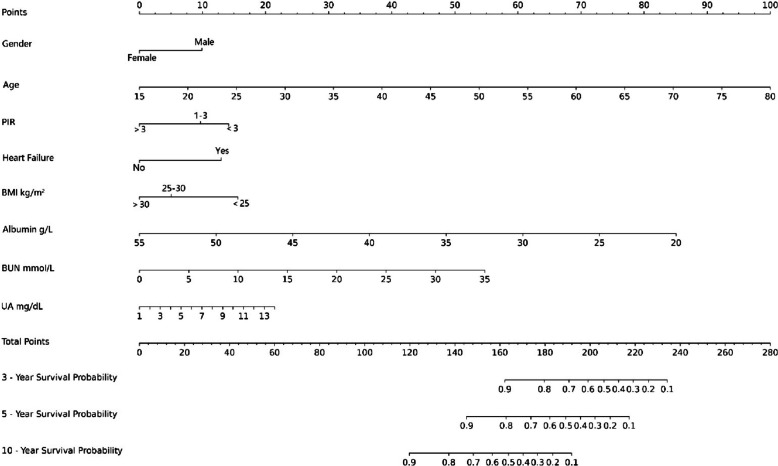
Nomogram of 3-year, 5-year,10-year survival probability in patients with diabetic nephropathy

Based on the results of the final model, we developed a nomogram for predicting the probabilities of all-cause mortality in patients with diabetic nephropathy at 3, 5, and 10 years (Fig. 3). The nomogram consists of several predictive variables, with each variable assigned a different weighted score in the diagram, and these scores carry distinct weightings. The final total score, obtained by summing individual scores, represents the 3-year, 5-year, and 10-year mortality risks, with a higher total score indicating a greater risk of all-cause mortality.

### Internal and external validation

**Fig. 4:**
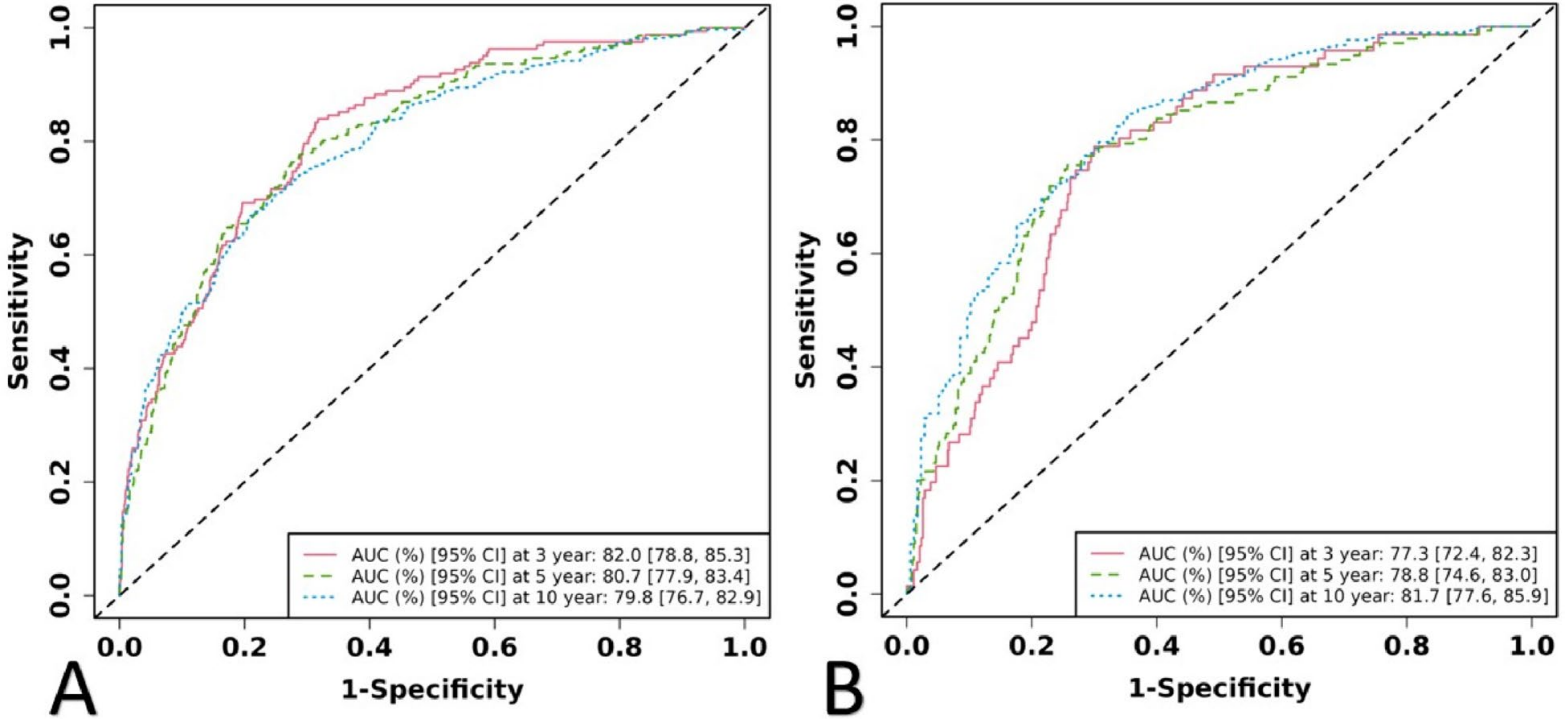
ROC curves for training and validation cohorts in the **A** and **B**

**Fig. 5:**
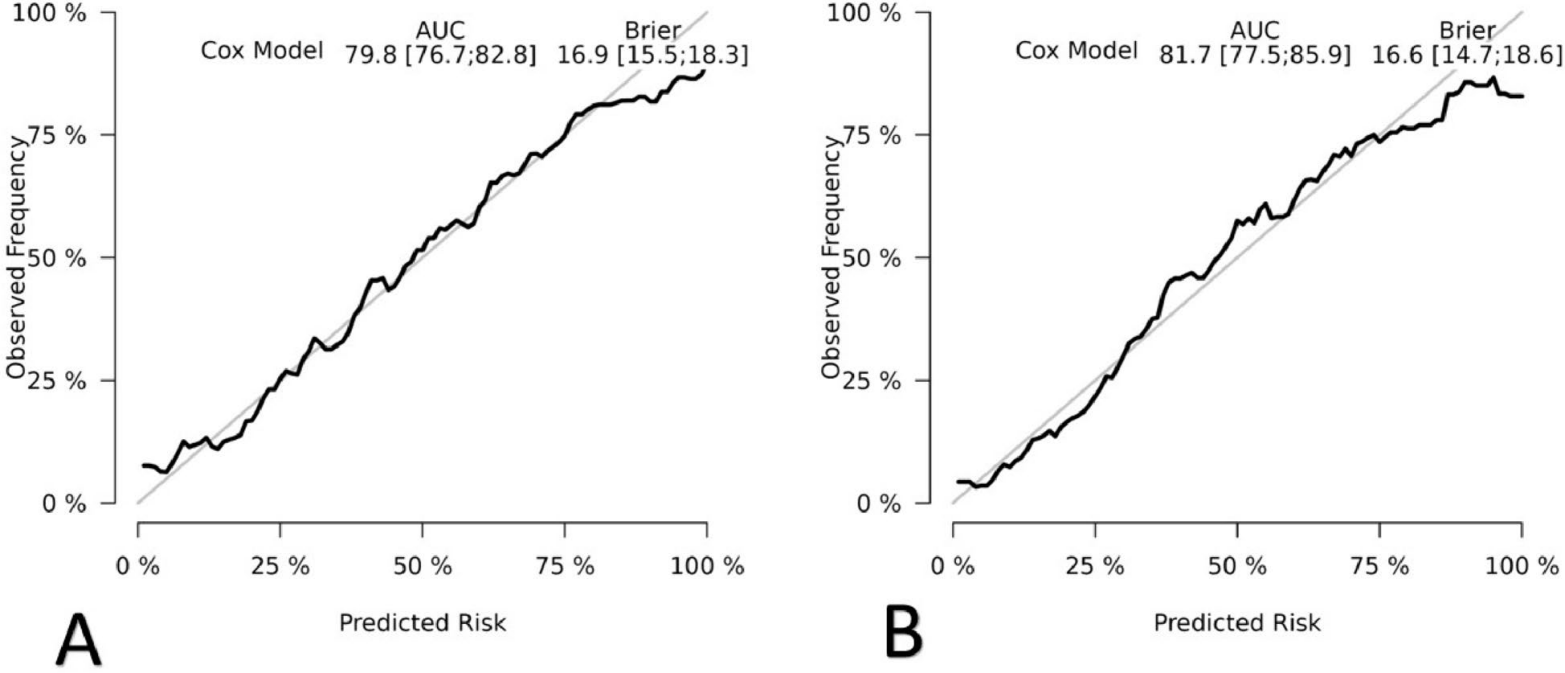
**A** Calibration curve for training cohort. **B** Calibration curve for validation cohort

Internal and external validation were conducted using ROC curves to assess the model’s performance. In the training cohort, the predictive models for 3-year, 5-year, 10-year mortality rates had AUCs of 0.820 (95% CI: 0.788–0.853), 0.807 (95% CI: 0.779–0.834), and 0.798 (95% CI: 0.767–0.829), respectively (Fig. 4A). In the validation cohort, the AUCs for the predictive models of 3-year, 5-year, 10-year mortality rates were 0.773 (95% CI: 0.724–0.823), 0.788 (95% CI: 0.746–0.830), and 0.817 (95% CI: 0.776–0.859), respectively (Fig. 4B), which indicates that our model has good stability and prediction accuracy. Calibration curves in both the training and validation groups demonstrated good consistency between the model’s predicted outcomes and actual results (Fig. 5A and B).

**Fig. 6:**
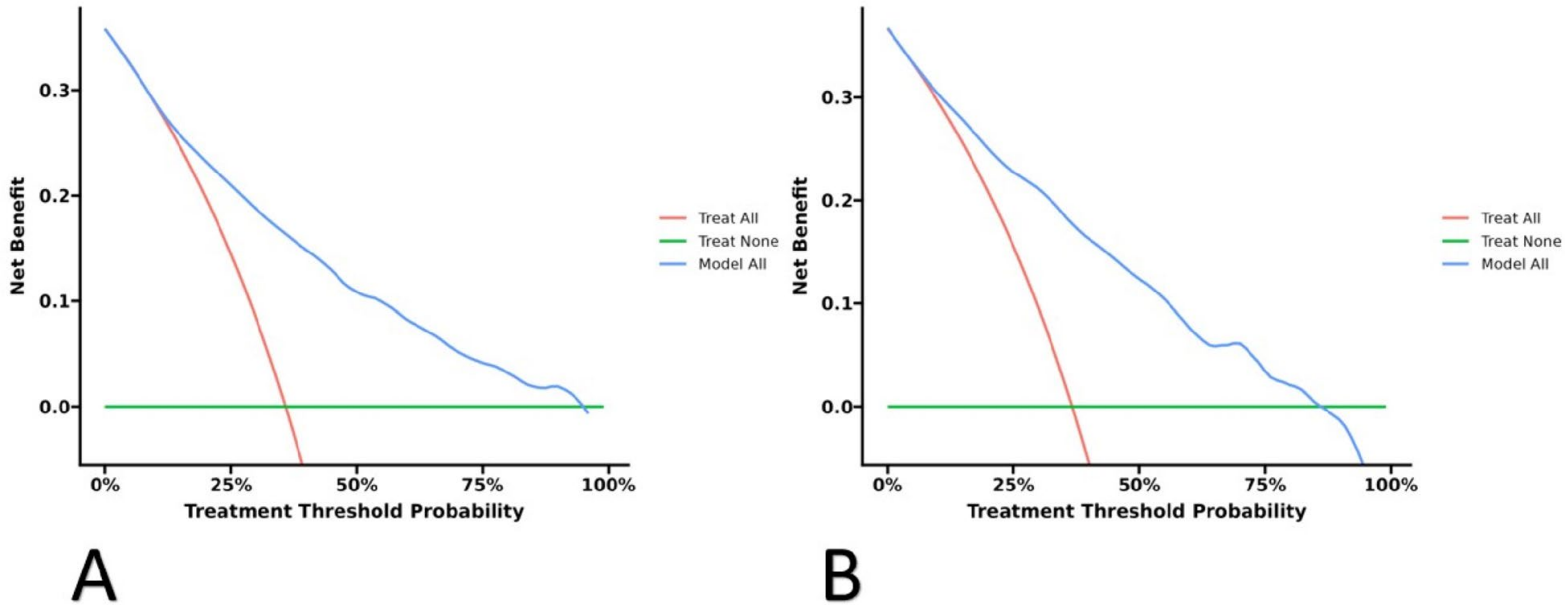
**A** DCA curve for the training cohort. **B** DCA curve for the validation cohort

The Fig. 6 displays the DCA curves associated with the nomogram for the training (Fig. 6A) and validation groups (Fig. 6B), indicating that the nomogram provides significant net benefits for clinical application based on its DCA curve. When the blue line is above both the “ALL” and “None” lines, it indicates favorable net benefit from intervention. The DCA curve assists in making better clinical decisions.

## Discussion

Utilizing NHANES data and follow-up results, we developed and validated a nomogram for predicting all-cause mortality in patients with diabetic nephropathy. This nomogram integrates patient characteristics with routine laboratory tests to estimate the all-cause mortality risk in these patients at 3, 5, and 10 years. Our study employed COX regression and LASSO regression, ultimately incorporating eight factors: gender, age, PIR, heart failure, BMI, albumin, blood urea nitrogen and serum uric acid. For male patients, older age, lower family income, concomitant heart failure, and underweight status, lower albumin levels, and higher levels of uric acid and blood urea nitrogen were associated with a higher risk of all-cause mortality. Previous research [16, 20–23] has consistently confirmed the varying impact of these factors on the survival of patients with diabetic nephropathy.

The global epidemic of diabetes continues to escalate, affecting both developed and developing countries. According to statistics, deaths related to vascular complications of diabetes saw a significant increase from 2000 to 2016, primarily driven by a rise in mortality associated with kidney complications [24]. The development and application of the nomogram can significantly aid doctors in identifying high-risk populations. With the growing population of diabetic patients, preventing complications and reducing mortality in patients with diabetic nephropathy are of paramount importance. Despite the use of inpatient data to construct the nomogram by Wang [25] in previous validations, many variables in their model require hospitalization. Currently, there is no predictive model for non-hospitalized patients, and this population deserves particular attention and awareness. Therefore, establishing an assessment tool to predict the all-cause mortality risk in patients with diabetic nephropathy is imperative. Our study aims to construct a prognostic model combining patient characteristics and simple laboratory tests to identify high-risk individuals and reduce the risk of premature death in patients with diabetic nephropathy. According to the internal validation of our research data, our nomogram effectively predicts the all-cause mortality risk in patients with diabetic nephropathy, with favorable calibration curve results. The DCA curve also suggests that intervention to a certain extent can increase the net benefit for patients. These findings confirm the performance of our nomogram.

Previous research has already identified and confirmed age as one of the factors influencing the overall mortality risk in patients with diabetic nephropathy [22]. In a prospective cohort study assessing the 2-year cardiovascular mortality risk in diabetic patients, age was found to be one of the factors contributing to cardiovascular mortality [20]. Similarly, in the context of machine learning predicting the incidence of diabetic nephropathy in diabetes patients, age showed a positive correlation with the risk of developing diabetic nephropathy [26]. Furthermore, in a study on overall mortality in type 2 diabetes patients, it was observed that deceased patients were generally older compared to those who survived [27]. This aligns with one of the predictive factors identified in our study, emphasizing that older patients bear a greater burden of overall mortality risk. Gender is also a significant factor in the relevant mortality of patients with diabetic nephropathy. Overall, male patients tend to have a higher risk of overall mortality compared to female patients. Wang [28] conducted a study on 2535 diagnosed participants with diabetes in NHANES from 1999 to 2018, and the results revealed that the risk of overall mortality and cardiovascular mortality was significantly higher in males than females. Similarly, it was observed that male diabetic nephropathy patients had a higher risk of both microvascular and macrovascular complications. Previous studies have consistently shown an increased risk of overall mortality and cardiovascular mortality in males compared to females [20, 22]. This difference may be attributed to variations in hormonal secretion between genders.

The Poverty Income Ratio (PIR) reflects the economic status of patients, and generally, individuals with better economic conditions are more likely to receive optimal treatment strategies. A nationwide survey in Korea, focusing on diabetic retinopathy and nephropathy screening in diabetic patients, revealed significant disparities in screening for diabetes-related complications due to socioeconomic inequality. Individuals with lower economic status were more likely to be at risk for early complications that went unnoticed [29, 30]. Similarly, a study in Germany, exploring the association between socioeconomic status and renal function in diabetic nephropathy patients, found that a lower socioeconomic status increased the risk of end-stage renal disease [31]. Additionally, patients with lower incomes may experience a lower quality of life, potentially further impacting disease management and treatment and increasing their risk of mortality [32].

Past research has found that being underweight in older adults increases the risk of age-related diseases and shortens life expectancy. Additionally, being underweight may serve as an indicator of frailty in the elderly [33, 34]. In a study investigating the factors contributing to all-cause mortality in early-stage diabetic nephropathy patients in Japan, underweight was identified as a risk factor for both all-cause mortality and ischemic heart disease mortality. In contrast, obesity showed no significant association with the risk of all-cause mortality. Underweight may serve as an intuitive indicator of malnutrition, ongoing diabetes progression, and mental health conditions in patients [35]. In a recent meta-analysis [36] examining the relationship between body weight and mortality in type 1 diabetes, the risk of death in the underweight group was 3.4 times higher than that in the normal weight group (95% confidence interval [CI], 1.67–6.85). Meanwhile, there was no significant difference in the risk of death between the normal weight group and the overweight group (HR, 0.90; 95% CI, 0.66–1.22) or the obese group (HR, 1.36; 95% CI, 0.66–1.22). A study investigating factors associated with mortality in patients undergoing hemodialysis found that maintaining or increasing BMI during hemodialysis could prolong the survival of patients. BMI may thus serve as one of the prognostic factors in the population of diabetic nephropathy patients [23].

Individuals with diabetes face an increased risk of developing heart failure and chronic kidney disease, and the presence of these comorbidities significantly elevates the incidence and mortality rates in diabetic patients [37]. A large multinational cohort study revealed that heart failure or impaired kidney function is associated with an increased risk of cardiovascular and all-cause mortality in diabetes patients, with HR of 2.02 (95% confidence interval [CI] 1.75–2.33) and HR 2.05 (95% CI 1.82–2.32), respectively. Moreover, diabetic patients with the combination of heart failure and impaired kidney function showed the highest risk of all-cause mortality (HR 3.14, 95% CI 2.90–3.40) [38]. Brain natriuretic peptide (BNP) serves as an objective marker for heart failure. In a Japanese study investigating the relationship between BNP levels and the prognosis of diabetic kidney disease, it was found that baseline BNP levels were associated with the prognosis of diabetic kidney disease after a follow-up period of seven years. Significantly different progression of diabetic kidney disease, cardiovascular events, and mortality risks were observed between individuals with low BNP and high BNP levels [39].

In contrast to the sociodemographic characteristics mentioned earlier, laboratory indicators provide a more objective reflection of patients’ physical condition and disease status. The use of biomarkers for diagnosis and prognosis has become increasingly common [40]. According to our study, certain laboratory parameters in diabetic kidney disease patients show correlations with all-cause mortality. In our study, there was a negative correlation between serum albumin levels and overall mortality in diabetic kidney disease patients. Albumin is a crucial indicator reflecting the nutritional status within the body. Higher serum albumin levels in diabetic patients are significantly associated with a lower risk of microvascular complications related to diabetes [41]. Research suggests that individuals with lower serum albumin levels in the diabetic kidney disease population are more prone to progress to end-stage renal disease (ESRD), leading to an unfavorable prognosis [42]. Uric acid and blood urea nitrogen (BUN) levels, on the other hand, show a positive correlation with all-cause mortality. The role of uric acid in diabetic kidney disease is not fully understood, but several studies suggest that sustained elevated uric acid levels may have a continuous impact on renal function in diabetic kidney disease patients, potentially leading to kidney damage and the occurrence of ESRD [21, 43]. BUN, as one of the metabolic indicators in the body, directly reflects the patient’s renal function status. Elevated BUN levels often indicate ongoing deterioration of kidney function.

Although this study utilized a large sample size from the NHANES database, with data controlled by professionals and follow-up measures in place, it still has some limitations. Firstly, the temporal constraints on data span might limit the applicability of this study to recent trends or changes in the risk of diabetic nephropathy and mortality. Secondly, the NHANES database primarily includes the American population, potentially lacking complete representativeness. These findings may not be applicable to certain subgroups or specific demographic populations, especially in low-income countries. Thirdly, limitations exist in the data collection methods, such as recall bias and the constraints of self-reported information, which may impact the accuracy of risk prediction. Fourthly, the NHANES data lack some important variables and detailed follow-up events, such as dynamic aspects of blood sugar control, the types of diabetes, treatment modalities for diabetes and diabetic nephropathy, and variables related to diet and exercise. These omissions could potentially influence the outcomes. Lastly, the absence of external validation on an independent dataset may limit the robustness and accuracy of these findings. These limitations underscore the necessity for future research to address these constraints and enhance our understanding of the mortality risk associated with diabetic nephropathy.

## Conclusion

The newly developed nomogram proves to be effective in predicting the all-cause mortality risk in patients with diabetic nephropathy, and it has undergone robust internal validation. This provides valuable information for the early identification of high-risk patients and facilitates effective clinical interventions.

## Data Availability

All data were included in NHANES database (https://www.cdc.gov/nchs/nhanes/index.htm). And the datasets used and/or analysed during the current study available from the corresponding author on reasonable request.
